# Alexithymia, but not autism spectrum disorder, may be related to the production of emotional facial expressions

**DOI:** 10.1186/s13229-016-0108-6

**Published:** 2016-11-11

**Authors:** Dominic A. Trevisan, Marleis Bowering, Elina Birmingham

**Affiliations:** 1 Faculty of Education, Simon Fraser University, 8888 University Drive, Burnaby, BC V5A 1S6 Canada; 2 Linguistics Department, Simon Fraser University, 8888 University Drive, Burnaby, BC V5A 1S6 Canada

**Keywords:** Autism, Alexithymia, Facial expressions

## Abstract

**Background:**

A prominent diagnostic criterion of autism spectrum disorder (ASD) relates to the abnormal or diminished use of facial expressions. Yet little is known about the mechanisms that contribute to this feature of ASD.

**Methods:**

We showed children with and without ASD emotionally charged video clips in order to parse out individual differences in spontaneous production of facial expressions using automated facial expression analysis software.

**Results:**

Using hierarchical multiple regression, we sought to determine whether alexithymia (characterized by difficulties interpreting one’s own feeling states) contributes to diminished facial expression production. Across groups, alexithymic traits—but not ASD traits, IQ, or sex—were associated with quantity of facial expression production.

**Conclusions:**

These results accord with a growing body of research suggesting that many emotion processing abnormalities observed in ASD may be explained by co-occurring alexithymia. Developmental and clinical considerations are discussed, and it is argued that alexithymia is an important but too often ignored trait associated with ASD that may have implications for subtyping individuals on the autism spectrum.

## Background

Autism spectrum disorder (ASD) is a complex neurodevelopmental disorder characterized in part by abnormalities in the social-emotional domain and atypical verbal and nonverbal communication [1]. Experimental and observational research shows that individuals with ASD have a variety of emotion processing abnormalities, including difficulties perceiving emotions in self and others, responding to others’ emotions in empathetic ways, and expressing emotions nonverbally to demonstrate empathy and to regulate social interactions [2]. However, there is wide heterogeneity in the severity of such difficulties within the ASD population, and there is emerging evidence to suggest that this heterogeneity may be driven by comorbid *alexithymia* [3]. Alexithymia is characterized by difficulties identifying and describing one’s emotions, lack of awareness that some physical sensations are due to emotions, an “external thinking” orientation that involves focus on external realities with limited self-reflective thought towards inner experience, and limited imagination and fantasy life [4, 5]. Like ASD traits [6], alexithymic traits are often measured continuously within the general population [7].

Using previously defined cut-off scores, alexithymia occurs in approximately 50 % of the ASD population [8], compared to approximately 13 % in the neurotypical population [9]. Despite the heightened co-occurrence of ASD and alexithymia, it is important to emphasize that they are independent constructs, as evidenced by the fact that 50 % of the ASD population appears to be unaffected by alexithymia, and many individuals in the general population, or with other clinical disorders, may have high levels of alexithymia. Additionally, recent fMRI evidence in ASD and neurotypical participants suggests alexithymia and ASD may have differing neurocognitive bases, as evidenced by the fact that ASD is associated with disruptions in brain networks associated with Theory of Mind functions but that alexithymia is associated with brain networks modulating affective processes (such as emotional awareness and empathy), but not associated with activation of ToM networks [10].

While ASD has been associated with impairments in recognizing others’ emotions and empathizing, there is evidence to suggest that not all individuals with ASD are impaired in these domains [11, 12]. Critically, recent studies suggest that alexithymia, not autistic traits, may be driving deficits in emotion recognition, empathy, and interoceptive accuracy in individuals with ASD, and it is possible that varying levels of alexithymia may account for the discrepant findings in the extant literature. For instance, when matched on levels of alexithymia, participants with ASD did not show deficits in emotion recognition tasks compared to controls [13]. Moreover, in a task that required participants to view stimuli of individuals experiencing pain, there were also no group differences in empathic insular response when ASD and control groups were matched for alexithymia [14]. Corroborating their findings, these studies examined the same variables continuously and found that alexithymic traits, but not autistic traits, predicted variability in the emotion recognition and empathy tasks. Another recent study found that controlling for alexithymia eliminates ASD and neurotypical group differences in interoceptive accuracy [15], a mechanism that is heavily implicated in our ability to experience and understand emotions [16]. These findings have lead Bird and Cook [3] to formulate the “alexithymia hypothesis,” which posits that individual differences in alexithymia may account for individual differences in various emotion processing abnormalities that are common (but not universal) within the ASD population.

Another component of emotion processing that may be relevant to the alexithymia hypothesis concerns the use of nonverbal body cues such as facial expressions to convey emotional information. Despite being a prominent diagnostic feature of ASD [1], the abnormal use of facial expressions in ASD has received little empirical attention. The sporadic research that has been done has shown that compared to neurotypical controls (or other non-ASD comparison groups), children with ASD are generally less expressive ([17–20]; cf, [21]), are less likely to naturally attend to and imitate others’ expressions [22–24], may display confusing or ambiguous facial expressions in which it is difficult to interpret what emotion they are expressing [18, 20, 25], and may display inappropriate facial expressions in which the expressed emotion does not match the feeling state of the individual or does not match the context of the situation [26]. However, the research on facial expression production in ASD has almost exclusively been motivated by an interest in finding group differences in facial expression production between ASD and control participants. Thus, there has been little consideration for the mechanisms at play that may contribute to abnormal facial expressions observed in ASD. Inspired by previous research in non-ASD populations, a central interest of the present study was to examine one such mechanism, *alexithymia*, in relation to facial expression production in children with and without ASD.

Indeed, a handful of studies have examined the link between alexithymia and nonverbal emotional expression in individuals *without* ASD [27–31]. Although results have not been entirely consistent, most studies found that higher levels of alexithymia are related to inhibited facial expression production. For example, Wagner and Lee [31] instructed participants to describe past positive and negative experiences and found that the salience of participants’ positive and negative facial expressions (as rated by trained raters) was negatively associated with their levels of alexithymia (such that higher levels of alexithymia were associated with less salient facial expressions). The same relationship was reported by Rasting et al. [29], who found that increased levels of alexithymia in clinical patients were correlated with reduced facial expressions during dyadic therapeutic interventions. Although the reason for this association is not well understood, the “conflict hypothesis” conjectures that alexithymic tendencies may develop as a mechanism to *defend* oneself against negative affect [29, 32, 33]. Thus, alexithymic individuals may consciously or subconsciously suppress their own facial expression displays in an effort to defend against negative affect and to avoid interpersonal conflict. This hypothesis would also predict the expression of *positive* affect to be unassociated with alexithymia, a possibility supported by the findings of Rasting et al. [29]. Alternatively, given that alexithymia is associated with impaired interoception [15], it may be that difficulties in emotional awareness (based on interoception) contribute to abnormal or diminished subsequent representation of these emotional states via facial expression or other motor indicators of emotion [34]. Adding further complexity, there is evidence to suggest that producing facial expressions may help to *induce* the experience of emotion in oneself [35, 36] which could aid accuracy in perceiving one’s own emotions. Thus, the relationship could be bi-directional such that alexithymia (perhaps via impaired interoception, as above) could impair one’s ability to produce facial expressions or that inaccurate or inhibited *external* expression of emotion (e.g., facial expressions) could lead to difficulties identifying and describing one’s own emotional states (e.g., alexithymia). Although blunted facial expression represents a prominent clinical feature of ASD [1], an untested prediction that follows is that the reduced displays of affect characteristic of ASD may be explained in part by heightened levels of alexithymia in this population.

### Present study

In the present study, we set out to determine how children with and without ASD may differentially produce facial expressions in response to emotional stimuli and whether alexithymia may contribute to diminished facial expressions. This study has the potential to advance our understanding of abnormal nonverbal emotional expression in ASD and examines the alexithymia hypothesis using an emotion processing abnormality that has yet to be examined (i.e., diminished production of facial expressions in response to emotional stimuli).

We used a novel approach to examine spontaneous facial expression production by showing participants various video clips designed to elicit a range of emotional reactions, while covertly recording their facial expressions with a webcam for subsequent analysis with automated facial expression analysis software. Previous research on spontaneous facial expressions has largely been explored by showing participants static or morphing images of isolated faces [24, 37, 38], which we suspected may not be stimulating enough to reliably parse out individual differences in facial expression production. Thus, the video stimuli we used (see Appendix) were carefully selected to be engaging and entertaining to the participants in order to produce substantial variance in participants’ facial expression production.

## Methods

### Participants

Thirty-four children—17 with an ASD diagnosis and 17 neurotypical controls—participated in this study. There were an equal number of boys and girls in each group. Participants were matched on IQ as measured by the Vocabulary and Matrix Reasoning subtests of the Wechsler Abbreviated Scale of Intelligence (WASI) [39]; the groups differed slightly but significantly on chronological age (see Table 1). The parent-report Autism Spectrum Quotient-Child Version (AQ) [40] was completed for all participants aged 7–11, and the AQ-Adolescent Version [41] was completed for participants aged 12–13. The child and adolescent versions of the AQ are nearly identical in content, but using differing scoring procedures (0–150 versus 0–50). For our purposes, the AQ-Child was scored in the same way as the AQ-Adolescent, on a scale from 0 to 50. (for justification, see [42]). As expected, the ASD group scored significantly higher on the AQ than the neurotypical (NT) group, *t*(31) = 4.84, *p* < .001.

**Table 1:** Means, standard deviations, ranges, and group differences of participant characteristics

	ASD (*n* = 17)	NT (*n* = 17)	*p*
Sex ratio M:F	13:4	13:4	–
Age	10.21 (1.78), 7.0–13.1	8.97 (1.30), 7.0–11.5	.027
AQ^a^	32.94 (8.85), 9–45	17.35 (9.61), 4–32	<.001
WASI—vocab	27.76 (10.65), 3–48	26.29 (7.00), 15–39	.637
WASI—matrix	17.29 (5.30), 7–25	17.12 (5.21), 7–24	.923

In the central columns, means are followed by standard deviations in parentheses, followed by ranges

*ASD* autism spectrum disorder, *NT* neurotypical, *AQ* autism quotient, *WASI* Weschler Abbreviated Scale of Intelligence

^a^All ASD participants scored 21 or higher on the AQ except one, who scored 9. Additionally, one neurotypical participant scored 32 on the AQ. We ran all analyses with and without these two participants treating them as potential outliers. The pattern of results was unaffected by their removal, and thus, we kept these participants in all analyses

Participants were recruited for one of two 1-day summer camps hosted by the Autism and Developmental Disorders Lab (ADDL) at Simon Fraser University (SFU). These day camps were designed to engage children in fun and educational activities related to social sciences, with dedicated time set aside for data collection. On subsequent Saturdays during the summer of 2015, identical camps were hosted—one for children with a diagnosis of ASD and one for children without ASD. Participants were recruited via a number of different methods. Email announcements of the camp were distributed to private and public schools in the area, and participants from the ADDL participant database were notified. In addition, information about the camps was posted on community websites, and fliers were posted on community billboards near SFU and surrounding cities.

Children in the ASD group received a standardized clinical diagnosis of ASD from an independent qualified pediatrician, psychologist, or psychiatrist associated with the provincial government-funded autism assessment network, or through a qualified private clinician in British Columbia (BC). All diagnoses were based on the Diagnostic and Statistical Manual of Mental Disorders (DSM-IV-TR) [43] and confirmed using the Autism Diagnostic Interview–Revised (ADI-R) [44] and the Autism Diagnostic Observation Schedule (ADOS) [45]. The province of BC has instituted standardized diagnostic practices for a diagnosis of ASD, as diagnosis is tied directly to substantial government funding in this province. All individuals must be diagnosed by ADOS and ADI-R trained clinicians who are required to use these tools as part of their assessment. Individuals who have been diagnosed in a different province or country are required to be re-diagnosed upon their arrival in BC using this protocol. In order to participate in the summer camp for children with ASD (at which data collection took place), parents were required to provide the camp coordinators with documentation from the BC government proving their children were diagnosed in BC using these standardized diagnostic practices. Inclusion criteria for the neurotypical control participants required no previous history of developmental, neurological, or psychiatric disorders.

### Materials

#### Video stimuli

The video participants viewed were a collation of ten short video clips from various children’s movies, documentaries, and home videos uploaded to Youtube.com. The clips were carefully selected to produce a wide range of emotional reactions (see details of clips in Appendix). Clips were separated by a 5-s blank screen. The entire video was 712-s (about 12 min) long.

#### Alexithymia

Alexithymia was assessed using the Children’s Alexithymia Measure (CAM), a parent-report measure designed to assess early childhood indicators of alexithymic tendencies [46]. The CAM consists of 14 items scored on a scale from 0 to 3. Total scores could range from 0 to 42, with higher scores representing higher levels of alexithymia. In the original validation study, the CAM’s 14 items were selected from a preliminary pool of 275, narrowed down by expert opinions of clinicians, and then again using factor analysis. A total of 224 parents of children who had experienced emotional trauma participated in that study [46]. To date, only one previous study [47] has used the CAM for research within the ASD population.

#### Autistic traits

In addition to reporting formal diagnoses, parents of all ASD and neurotypical participants completed the 50-item parent-report AQ-Child or AQ-Adolescent [40, 41] so that autistic traits could be assessed continuously. The AQ was scored on a range from 0 to 50, with higher scores indicating higher levels of autistic traits. This measure is not a diagnostic tool and is used primarily for research purposes. The AQ assesses social and nonsocial characteristics of autism relating to social skills, communication skills, attention to detail, imagination, and tolerance of change.

#### Intelligence

Intelligence was estimated with the WASI [39], which includes two subtests of nonverbal intelligence (Block Design and Matrix Reasoning) and two subtests of verbal intelligence (Vocabulary and Similarities). Due to time constraints of the camp, only the Vocabulary and Matrix Reasoning subtests were administered. The Vocabulary subtest assesses abilities related to word knowledge and verbal concept formation by testing participants’ ability to provide word labels for objects presented to them and their ability to provide definitions of words that are presented visually and orally. The Matrix Reasoning subtest assesses abilities related to spatial reasoning, fluid intelligence, and perceptual organization by requiring participants to view a series of incomplete matrices and complete them by selecting the correct response option.

#### Facial expression analysis technology

Facial expressions were analyzed using iMotions’ facial expression analysis technology called “FACET” [48], which uses the facial action coding system (FACS) [49, 50] to estimate the degree to which each of seven basic emotions (joy, surprise, sadness, disgust, contempt, anger, and fear) is being expressed at any given time frame (1/30th of a second). FACS has been the gold star facial expression coding system for decades and has proven to be a useful and reliable method [51, 52]. Traditionally, FACS coding has been done by trained experts who required hundreds of hours of training to become “FACS certified” [49]. However, coding by hand has proven to be quite onerous and overwhelming and is particularly difficult when coding dynamic (transitioning) or blended expressions (combinations of multiple emotions) [53]. FACET is particularly useful in this regard, as it automatically codes expressions based on a library of thousands of expert human coders. While FACET is in need of an extensive validation study, its academic predecessor, the Computer Expression Recognition Toolbox (CERT), from which FACET’s technology was derived, has demonstrated strong psychometric properties, as evidenced by its ability to correctly detect emotions from standardized facial expression stimuli [54].

The basic emotions estimated by FACET can also be collapsed into global categories of positive, neutral, and negative emotions. FACET reports the probability that each emotion is being expressed using a base 10 logarithmic likelihood estimate. For example, a joy threshold value of −1 (i.e., 10^−1^) indicates a probability of 10:1 that joy is *not* being expressed, whereas a value of 2 (i.e., 10^2^) indicates a probability of 100:1 that joy *is* being expressed. Within the iMotions interface, researchers have the opportunity to set their own threshold values for FACET. Based on the recommendations of an iMotions representative (A. Viramontes, personal communication, October 26, 2015), we set our threshold value at .5, which translates to a probability of 10^5^ (roughly 3.16). Our dependent variable was the percentage of time participants expressed various emotions during the 712-s video. Thus, a joy percentage value of 22 % can be interpreted as “joy was expressed at least 3.16 times more likely than not during 22 % of the video.”

### Procedure

Before the camp, parents completed and submitted a number of forms and questionnaires for research and camp purposes, including those analyzed in the present study. All experimental data were collected during camp activities. The camp was separated into seven different sessions during a 6-h period (with lunch and other breaks throughout). Each session lasted approximately 40 min. The present study was included in a session along with another research activity (unrelated to the present study). As such, 20 min of the session was devoted to the present study. Groups of four to eight participants completed the task at one time, with participants separated by cardboard dividers to prevent distraction. Each participant watched the video clips (presented using Quicktime Player 10.4) on their own laptop computer (13 or 11” MacBook running OSX 10.11.3) with headphones. Participants’ facial expressions while watching the clips were covertly recorded with a built-in webcam.

### Data preparation

The video stimuli failed to produce substantial facial expressions for certain emotions (especially disgust and surprise), and the distributions of individual emotions (except for joy) were sometimes non-normal due to floor effects. To increase statistical power, and thus normalize the distributions of emotions, we did not conduct analyses with individual emotions. Instead, we examined three categories automatically calculated by FACET; *negative* (comprising anger, fear, surprise, sadness, disgust, and contempt); *positive* (includes only the expression of joy); and *neutral* (no emotional expression). We also created a variable that combined negative, positive, and neutral expression, after first transforming the neutral expression variable by subtracting each threshold percentage from 1 in order to maintain a consistent directionality with the negative and positive emotion categories. This composite variable was created mainly to serve as the sole dependent variable in a subsequent regression analysis, in order to reduce the needed number of statistical tests.

Before conducting any analyses, we assessed normality of the AQ, CAM, and each of the expression category variables with visual inspection, aided by Tabachnick and Fidell’s (2007) recommendation to divide skewness by its standard error and assess non-normality based on the calculated z-score [55]. The AQ, CAM, and two of the three composite variables (negative and positive emotion) were deemed to have acceptable normality. Visual inspection of the neutral expression variable revealed a negative skew, and the observed z-score was 2.84 suggesting the distribution was non-normal. We transformed the neutral variable to normalize the distribution using a two-step transformation process [56]. All following analyses examining neutral expression utilize the transformed neutral variable.

Each of the variable categories are calculated simultaneously and independently by FACET, such that any combination of emotions can be detected at any given time point. For example, while watching Shrek brush his teeth with the guts of a worm, he squeezed onto his toothbrush (see Appendix); participants may respond to the clip with both joyful and disgusted facial expressions. In addition to detecting expressions of joy and disgust, FACET also usually detects *neutral* when expressions are not very intense. Intercorrelations among positive, negative, and neutral composite variables revealed no statistically significant associations (all *p*s >.20), suggesting that they are each tapping into independent variables.

## Results

### Correlations between facial expression production, ASD, and alexithymic traits

As a first step to testing the alexithymia hypothesis [3] in the context of our study—that alexithymia better predicts variance in facial expression production than autistic traits—we examined Pearson’s *r* correlations between the facial expression production variables and alexithymic and ASD traits, separately. Alexithymic traits (as measured by the CAM) were inversely associated with negative expression, such that a higher level of alexithymia was associated with a lower proportion of time for negative expressions, *r* = −.37, *p* = .030. CAM scores were also *positively* correlated with neutral expression, *r* = .43, *p* = .012, such that a higher level of alexithymia was associated with a higher proportion of time for neutral expression. CAM scores were not, however, associated with positive expression at a statistically significant level, *r* = −.13, *p* = .459. In addition, CAM scores were correlated with the composite variable in the expected direction, such that a higher CAM score was associated with less expression overall (and more neutral expression), *r* = −.49, *p* = .002. Next, we examined correlations between AQ scores and the same facial expression variables. Three of the four correlations were statistically nonsignificant: negative, *r* = .01, *p* = .962; positive *r* = −.03, *p* = .857; composite variable, *r*, = − .12, *p* = .491. The one exception was a significant association with neutral, *r*(32) = .38, *p* = .031. However, as the alexithymia hypothesis would predict, when the effects of alexithymia were partialled out, the relationship was all but eliminated; partial *r* = .04, *p* = .812.

### Multiple regression analysis predicting facial expression variance

We next directly examined our main hypothesis, that alexithymic traits are a better predictor than autistic traits of variance in facial expression production. To this end, we conducted a three-step, forced entry hierarchical regression analysis following the procedures of Cook et al. [13], using the composite facial expression variable as the dependent variable (see Table 2). In the first step, we entered all potential covariates including age and sex, as well as the Vocabulary and Matrix Reasoning subtests of the WASI to account for IQ. This model did not produce a statistically significant effect, *F*(4, 28) = .596, *p* = .669, *R* ^2^ = .078. In the second step, we added AQ scores to the model to see whether autistic traits predicted variance in facial expression production beyond the effects of the covariates. Again, the model was not statistically significant, *F* change (5, 27) = .200, *p* = .659, adding a negligible .007 *R* ^*2*^ change beyond the effects of model 1. Finally, CAM scores were entered into the model, producing a statistically significant influence beyond the effects of autistic traits and the covariates, *F* change (6, 26) = 15.842, *p* < .001, adding .346 *R* ^*2*^ change to the model. The final model explained 43.2 % of the variance in facial expression production. Approximately 34.6 % of the variance in facial expressions was explained by alexithymic traits in step 3, while the remaining 8.6 % in variance was explained by the combined, nonsignificant effects of the covariates and ASD traits in steps 1 and 2.

**Table 2:** Model summary of hierarchical regression analysis

Model	*R*	*R* ^2^	Adjusted *R* ^2^	SEE	*R* ^2^ change	*F* change	Sig. *F* change
1	.280^a^	.078	−.053	38.12	.078	.596	.669
2	.292^b^	.085	−.084	31.68	.007	.200	.659
3	.657^c^	.432	.300	31.07	.346	15.842	<.001

*a* predictors: age, sex, IQ; *b* predictors: age, sex, IQ, AQ; *c* predictors: age, sex, IQ, AQ, CAM

*SEE* standard error of the estimate

One consideration is that the two main independent variables of interest—alexithymic and ASD traits—were moderately correlated, *r* = .46, *p* = .007. Thus, the results of the regression analysis may be affected by order of entry and multicollinearity. We therefore ran an additional analysis, this time entering alexithymic traits in step 2 and ASD traits in step 3. The relative contributions of either variable were similar to that of the previous analysis. The only significant contribution came from alexithymic traits in step 2, *F* change (5, 27) = 14.095, *p* < .001, *R* ^*2*^ = .316, while ASD traits were, again, nonsignificant, *F* change (6, 26) = 1.694, *p* = .204, *R* ^*2*^ = .037.

### Group differences in alexithymic traits

Next, we examined differences between ASD and NT groups in their degree of alexithymic traits as measured by the CAM. As predicted, an independent samples *t* test revealed that the ASD group scored higher on the CAM (*M* = 17.29, SD = 8.55) than the NT group (*M* = 8.41, SD = 8.88), indicating higher levels of alexithymic traits, *t*(32) = 2.97, *p* = .006, Cohen’s *d* = 1.02.

### Group differences in facial expression production

Because the ASD group scored more than one standard deviation above the neurotypical group on alexithymia scores, and because we found the higher alexithymia scores are associated with reduced expression production, a clear prediction is that the ASD group would be significantly less expressive than the NT group. To test this possibility, we report the results of a two-group, between-subjects Multiple Analysis of Variance (MANOVA) with diagnosis as the independent variable and neutral, positive, and negative emotion production as the dependent variables. The composite variate was not significantly affected by diagnosis but approached significance, Wilks’ lambda = .808, *F*(3, 30) = 2.115, *p* = .090, partial eta squared = .192. Univariate effects are reported in Table 3. The only significant difference was that the ASD group expressed more neutral expression in the expected direction. Group differences in positive and negative emotion were nonsignificant. This pattern reflects only partial support for the alexithymia hypothesis, in that the ASD group produced more neutral expression than the NT group (despite being statistically equivalent on negative and positive expression production).

**Table 3:** Group differences in emotional expression

	NT (*n* = 17) Mean (SE)	ASD (*n* = 17) Mean (SE)	Partial eta squared	*p* value	Observed power
Positive	11.41 (12.11)	8.41 (8.51)	.021	.409	.128
Negative	47.81 (21.07)	60.19 (31.79)	.053	.191	.255
Neutral	.481 (0.10)	.601 (0.19)	.150	.024	.633

The means for negative and positive emotion represent the percentages of time each emotion category which was detected by FACET at the 10^5^ threshold level. The neutral variable was transformed to normalize the distribution and the means do not reflect percentages

## Discussion

The main purpose of this study was to advance previous work on the “alexithymia hypothesis” which demonstrated that alexithymia accounts for deficits in emotion recognition abilities, empathy, and interoceptive accuracy in adult samples of participants with ASD. To extend their findings to other components of emotion processing, we examined the effects of alexithymic and autistic traits on the production of spontaneous facial expressions in children with and without ASD, as they watched emotionally salient video stimuli. Consistent with Bird and Cook’s “alexithymia hypothesis” [3], two hierarchical multiple regression analyses confirmed that alexithymic traits, but not autistic traits, predicted variance in participants’ facial expression production.

As described in the introduction, children with ASD have been shown to produce diminished facial expressions relative to neurotypical children and are less likely to reciprocate other people’s expressions in real-world or experimental settings [18, 20, 22–24]. Our study provides support for Brewer et al.’s [25] prediction that alexithymic traits may be contributing to reduced facial expression production characteristic of ASD. In Brewer et al.’s study, participants with and without ASD attempted to recognize facial expressions posed by neurotypical individuals and individuals with ASD. They found that both neurotypical participants *and* participants with ASD were less able to infer the emotions expressed by posers with ASD compared to the expressions of the NT posers, providing support for the notion that individuals with ASD produce atypical, less recognizable facial expressions. While they found that alexithymic and ASD traits were highly correlated in their sample (*r* = .667), they did not report an association between alexithymia and facial expression production.

Why is alexithymia associated with diminished facial expression production? As discussed in the introduction, the *conflict hypothesis* proposes that highly alexithymic individuals may suppress their own facial displays to defend against negative affect and to avoid conflict [29, 32, 33]. This explanation would suggest that alexithymic individuals might unconsciously suppress their unfavorable negative emotions such as anger or sadness in an effort to distance themselves from distressing inner emotions and to mitigate potential external conflict. Such a possibility would also predict the expression of non-distressing emotions (e.g., joy) to be *unaffected* by alexithymia. Our data support this notion, as we observed in our sample that higher alexithymic traits were associated with less negative expressions, but they were not significantly associated with the amount of positive expression. This result is consistent with Rasting et al.’s [29] findings that the expression of negative emotion, but not positive emotion, was correlated with alexithymia in clinical patients during therapeutic interventions.

It is important to consider that a relative lack of facial expressions does not necessarily indicate a lack of physiological emotional arousal; that is, bodily signals of emotion may be present even in the absence of facial displays of emotion. It has been suggested that alexithymia is characterized by a deficit in interoceptive accuracy despite the fact that behavioral and autonomic reactivity are present [15, 57]. These considerations may help to explain the previously reported relationship between alexithymia and *emotion regulation* [58]. Not being consciously aware of one’s emotions as they arise, actively suppressing one’s emotions or refusing to acknowledge that one is experiencing negative emotions would inhibit one’s ability to regulate emotions as they increase in intensity—shedding light on the seeming paradox by which alexithymic individuals are prone to displaying minimal nonverbal emotional expression most of the time but are also prone to intense emotional outbursts [4, 27, 46]. As emotion regulation issues—and ensuing emotional outbursts—are common in ASD [59–61], future examinations of the “alexithymia hypothesis” may find that alexithymia is a major contributor to emotion regulation difficulties in people with ASD. Support for this idea comes from research that shows that compared to neurotypicals, participants with ASD are more likely to use *suppression* (e.g., “*I keep my emotions to myself*” and “*When I am feeling negative emotions, I make sure not to express them*”) as an emotion regulation strategy [60] as measured by the Emotion Regulation Questionnaire [62]. Thus, it appears that a relative disposition towards ignoring or suppressing one’s emotions compounded by poor emotional insight (e.g., alexithymia) may contribute to emotion regulation problems in ASD [59].

### Revisiting the alexithymia hypothesis

When we examined the alexithymia hypothesis using correlational and regression analyses, we observed a clear pattern of results in support of the hypothesis such that alexithymia, but not ASD traits (measured by the AQ), were associated with facial expression production. When we examined the alexithymia hypothesis at the group level of diagnosis, we found that that the ASD group expressed significantly more neutral expression than the neurotypical group, which would be expected considering the ASD group had higher levels of alexithymia. However, no group differences emerged on levels of negative expression. Similarly, no group differences were observed in level of positive expression, but this is consistent with our correlational analyses and the “conflict hypothesis” that would predict positive expression production to be unassociated with alexithymia. In sum, the groupwise comparisons reveal a murkier pattern of results than what was found in the regression analyses. However, these results should be interpreted with caution as the analyses were underpowered as indicated in Table 3. In addition, it is quite possible that there are other factors besides alexithymia that contribute to differences in facial expression production in the ASD population that were unaccounted for in the present study, and future research is needed to uncover these additional mechanisms.

The “alexithymia hypothesis” is intuitively appealing due to its simplicity and robust implications. Bird and Cook [3] argue that, given the wide heterogeneity in emotion processing abnormalities observed in the ASD population, it may be useful to create diagnostic subtypes of ASD reflecting the presence or absence of alexithymia. This idea is certainly worthy of continued exploration. However, one of the limitations of the alexithymia hypothesis is that it fails to explain why individuals on the autism spectrum are much more likely than the neurotypical population to possess a strong disposition for alexithymia. Alexithymia and ASD are independent constructs, but research is needed to uncover the reasons for why ASD and alexithymia often co-occur. Investigating potential differences between alexithymia in ASD and non-ASD populations may help shed light on this issue, in addition to conducting rigorous developmental and longitudinal research [2, 3]. Indeed, the present study is only the second to examine alexithymia in children with ASD, and no studies to date have examined alexithymia longitudinally in this population. Further, while the present research, consistent with past studies [13–15], shows that alexithymia “predicts” other emotional symptoms in the statistical sense, future research with more sophisticated research designs including longitudinal and structural equation modeling techniques are needed to untangle the relative causal associations among these variables.

Alexithymia is thought to represent an impaired affective representation system, in which the alexithymic individual may be aware they are upset but not able to identify which emotion they are experiencing [34], perhaps in part due to impaired interoceptive accuracy. Lacking the ability to differentiate internal affective states could inhibit one’s ability to associate affective internal states with perceptual cues that represent the emotional states of others [34]. For example, an fMRI study showed that in individuals with ASD, alexithymia is associated with impairments in the ability to report one’s own emotions, as well as with self-reported empathy, and that this relationship may be due to reduced activity in the anterior insula [63]. This complex interplay between emotion processing in self and other may explain why alexithymia is accounting for atypical emotion recognition, empathy, emotional awareness, and here—expression production—in ASD. By this line of reasoning, alexithymia may contribute to deficits in paired associate learning in which internal representations of affective states are not conditioned to be paired with external representations. Thus, in the context of the present study and past research, alexithymia may contribute to deficits in external representations of emotions in others (recognizing others’ facial expressions) or in the self (representing one’s own emotions with accurate and salient facial expressions).

Deficits in emotion recognition and empathy have been associated with ASD, but recent evidence suggests alexithymia accounts for these effects [13, 14]. As Bird and Viding [34] describe, empathy involves an “affective coding” process by which an observer’s internal affective state matches that of an object through specialized perceptual systems that are sometimes beneath conscious awareness. Alternatively, emotion recognition requires a “cognitive coding” process by which individuals are consciously aware of and can assign an appropriate label (e.g., *anger*) to another’s emotional state. This cognitive encoding process could be aided by the affective coding process [64], by which individuals arrive at emotion attributions by “simulating” others’ emotions in oneself. However, in many social situations, an observer’s emotional state may be very different than that of another. In such cases, individuals must selectively *attenuate* one’s interoceptive signals in order to prevent interference with the decoding process. [65]. Quatrocki and Fristron review evidence that the oxytocin system is responsible for selective attenuation of interoceptive signals [65], which may help explain why oxytocin administration improves emotion recognition abilities to a greater extent for individuals who are more highly alexithymic and less emotionally expressive [66].

### Utility of the CAM for ASD research

Although not a main aim of this study, we examined group differences in levels of alexithymic traits between children with and without an ASD diagnosis. We were initially skeptical of the CAM’s utility in the ASD population given the strong verbal component of the items. For example, items on the CAM include “Has trouble finding words or getting words out when talking about his/her own feelings,” “Verbal expressions of feelings do not match non-verbal expressions of feelings,” and “Says “I don’t know” when asked why he/she is upset.” In other words, if participants with ASD scored higher than the neurotypical group on the CAM, would these differences be better explained by *true* differences in alexithymic traits or by deficits in verbal ability? We found that the ASD group in our sample scored more than one standard deviation higher on the parent-report Children’s Alexithymia Measure (CAM) than the neurotypical control group. Importantly, the two groups were matched on measures of verbal (and nonverbal) IQ, and verbal IQ was not significantly associated with alexithymia, indicating the higher CAM scores in the ASD group were not explained by lower verbal ability. This finding replicates the large group differences in CAM scores between children with and without ASD reported by Griffin et al. [47] and complements studies showing higher rates of alexithymia in adults with ASD [8, 67].

The present study is just the second to examine alexithymia in children with ASD using the CAM. In addition to using the CAM, Griffin et al. [47] assessed alexithymia using the Children’s Alexithymia Questionnaire-Self Report (CAQ-SR; [68]). While both measures were sensitive enough to demonstrate higher levels of alexithymia in the ASD participants, correlational analyses revealed no relationship between the two measures indicating that children and their parents may be using different sources of information or that the content of the respective measures is highly dissimilar. Self-report measures of alexithymia have been criticized because, by definition, highly alexithymic individuals may lack the awareness to accurately assess their own levels of emotional awareness [3]. On the other hand, parent-report measures have the disadvantage of relying on parents to accurately assess their own children’s emotional awareness by indicators such as how often they express and talk about their emotions verbally, which may not accurately reflect how emotionally aware their children actually are. Increased research is needed to evaluate the relative strengths and weaknesses of self and other-report measures of alexithymia in children with ASD.

Given that our groups were matched on levels of verbal ability, we can tentatively conclude that the large group differences in alexithymia were not explained by differences in verbal ability. However, future research may benefit from developing measures that specifically assess emotion-word vocabulary (rather than domain-general vocabulary) as a control variable. It is possible that children with high levels of alexithymia have normal verbal intelligence and vocabulary knowledge but lack sufficient word labels to describe emotional states due to deficient emotional understanding. While continued research is needed to verify the appropriateness of the CAM for research in the ASD population, our initial findings seem promising. We do, however, caution that the ASD participants in our sample had average to above average IQ. It remains a likely possibility that the CAM would not be an appropriate tool for use in lower functioning children with ASD as it would be difficult to distinguish whether high scores on the CAM reflect true alexithymic tendencies or possible language or communication delays.

### Limitations

Our sample size was small, limiting the power to detect group difference in expression production and potentially producing unreliable effects in the between groups analysis. As a result, clear conclusions about whether alexithymia contributes to abnormal facial expression in the ASD population remain lacking. Future research on this topic will benefit from matching ASD and neurotypical groups on alexithymia (e.g., [13]) to shed more light on this issue. It is also possible that reactions to videos do not authentically capture how expressive participants are in real-world settings. Indeed, many of the studies on facial expressions in ASD reviewed in this article examined facial expression use during naturalistic social interactions. In a similar vein, it is possible that individuals with ASD, in comparison to neurotypicals, display markedly reduced expression only when regulating social behavior (i.e., during social interactions) but are not less expressive when passively viewing stimuli. This possibility offers an interesting possibility for future research.

In addition, our small sample size precluded the possibility of examining within group correlations. In fact, to our knowledge, virtually all studies that have examined alexithymia in ASD have utilized small samples (*N* < 100). Large-scale studies are urgently needed to more accurately examine how much of the ASD population displays high levels of alexithymia and to further our understanding of the full range of emotional processing differences that may be accounted for by alexithymia within this population. In addition, while the video stimuli we used were effective in that they yielded wide variance in facial expression production in our participants, the ways in which participants express emotions in response to videos may not be reflective of how they express emotions in real-world settings. Future research should replicate the present study in more ecologically valid contexts to explore how facial expressions may be used for empathizing and understanding others’ emotions.

Mentioned earlier, in addition to diminished facial expressions, individuals with ASD also tend to display *abnormal* facial expressions, which could not be directly assessed from the facial expression analysis software we used. Future research would benefit from finding ways to objectively measure abnormal facial expressions. For example, Ekman and Friesen [49] and Ekman et al. [50] have identified 46 specific action units controlled by facial muscles (e.g., “cheek raise” and “nostril dilation”) that combine in various ways to represent each of the basic emotions. An ambitious but worthwhile aim of future research will be to identify combinations of action units that are common in ASD, but atypical in the general population, to identify the precise combinations of facial movements that contribute to abnormal facial expressions in the ASD population. In addition, Brewer et al. [25] recently introduced a clever paradigm whereby atypical expression production in ASD was measured by how well others could interpret the intended emotion conveyed by facial expressions

A final limitation is that we only assessed alexithymia using a parent-report questionnaire as time limitations of the camp did not allow us to collect self-report data from the participants. Future research should continue to utilize self- and other-report measures.

## Conclusions

The main crux of our study explored the relationship between facial expression production and alexithymic traits in children with and without ASD. Our finding that alexithymic traits, but not autistic traits, predicted how expressive participants were in response to emotionally charged video stimuli adds to a growing body of literature that alexithymia seems to account for heterogeneity of emotional processing abnormalities in this population. Importantly, our data suggest that alexithymia may be contributing to a particular diagnostic criterion of ASD related to reduced facial expression production. In addition, we found large group differences in alexithymia between the ASD and neurotypical participant groups in our sample, corroborating the findings of Griffin et al. [47] that alexithymic traits are prevalent during childhood in individuals with ASD.

We urge that significantly more research and clinical attention should be devoted to the alexithymia construct in the ASD population. If further research verifies that alexithymia drives heterogeneity in emotion processing abnormalities, alexithymia will likely continue to emerge as an important consideration for researchers and clinicians who work with people with people on the autism spectrum. Moreover, if future research corroborates our findings, reduced facial expression may be identified as a potential indicator of alexithymia that could be used to aid children’s emotional development. For example, parents, educators, and other caregivers may play an important role in fostering their children’s emotional understanding by encouraging verbal and nonverbal emotional expression and by providing them feedback that helps children understand *what* emotions they are experiencing, *why* they are experiencing them, and offer strategies to help regulate negative emotions [57]. Future research would benefit from exploring these issues further in naturalistic child-caregiver interactions. Measures of alexithymia such as the CAM are not only useful for research purposes but may be used to aid clinical assessment and target optimal interventions in this population.

## Appendix

**Table 4:** Description of video stimuli

Name of video	Scene description	Primary emotion	Secondary emotion(s)	Clip duration
Tangled	Rapunzel discovers she is the lost princess, kidnapped at birth by Mother Gothel (who Rapunzel believed to be her real mother) to exploit Rapunzel’s powers in order to stay young. During the scene, Rapunzel confronts Mother Gothel and a heated argument ensues.	Anger	Fear, contempt	114
Inside Out	The character, Riley, and her personalized emotion character, Disgust, make multiple expressions of disgust as Riley swats a fly with a paper and peers underneath.	Disgust	NA	25
Monsters Inc.	The character, James P. Sullivan, has to say goodbye forever to his young friend Boo.	Sadness	NA	38
All Dogs Go To Heaven	The character, Charles B. Barkin, encounters all sorts of evils in the depths of hell during a nightmare.	Fear	NA	87
Shrek	Introductory scene showing the character, Shrek’s, unhygienic living conditions.	Disgust	Joy	82
A Conversation with Koko	The narrator describes a sad event where Koko’s beloved pet cat, Allball, was tragically killed by a car.	Sadness	NA	120
“Tennis Cats” (Youtube)	Four kittens move their heads in unison as their gaze follows the movement of an unknown object from behind the camera	Joy	NA	23
“Best of BBC Talking Animals” (Youtube)	Parodies of a nature show that dubs human voices over the clips as if the animals are talking to each other	Joy	NA	98
“Hysterical Bubbles!—laughing baby (Youtube)	A baby laughs hysterically as a dog gleefully pops bubbles with her mouth blown by the baby’s mother.	Joy	NA	27
Emotional Baby! Too Cute! (Youtube)	A baby smiles and cries in response to her mother singing a sad song.	Joy	Sadness	53

We have confirmed under the provisions of *CCH Canadian Ltd. v. Law Society of Upper Canada* (2004) that our use of portions of copyrighted material for the purposes of research is both legal and ethical [69]

## Abbreviations

AQ: Autism spectrum quotient
ASD: Autism spectrum disorder
CAM: Children’s Alexithymia Measure
NT: Neurotypical
WASI: Wechsler Abbreviate Scale of Intelligence
